# *In vivo* bioluminescence imaging of *Escherichia coli* O104:H4 and role of aerobactin during colonization of a mouse model of infection

**DOI:** 10.1186/1471-2180-12-112

**Published:** 2012-06-20

**Authors:** Alfredo G Torres, Roberto J Cieza, Maricarmen Rojas-Lopez, Carla A Blumentritt, Cristiane S Souza, R Katie Johnston, Nancy Strockbine, James B Kaper, Elena Sbrana, Vsevolod L Popov

**Affiliations:** 1 Department of Microbiology and Immunology, University of Texas Medical Branch, Galveston, TX, 77555-1070, USA; 2 Department of Pathology, Sealy Center for Vaccine Development, University of Texas Medical Branch, Galveston, TX, 77555-1070, USA; 3 Bacteriology Laboratory, Instituto Butantan, São Paulo, 05503-900, Brazil; 4 Enteric Diseases Laboratory Branch, Centers for Disease Control and Prevention, Atlanta, GA, 30333, USA; 5 Department of Microbiology and Immunology, University of Maryland School of Medicine, Baltimore, MD, 21201, USA

## Abstract

**Background:**

A major outbreak of bloody diarrhea associated with Shiga toxin-producing *Escherichia coli* O104:H4 occurred early in 2011, to which an unusual number of hemolytic uremic syndrome cases were linked. Due to limited information regarding pathogenesis and/or virulence properties of this particular serotype, we investigated the contribution of the aerobactin iron transport system during *in vitro* and *in vivo* conditions.

**Results:**

A bioluminescent reporter construct was used to perform real-time monitoring of *E. coli* O104:H4 in a mouse model of infection. We verified that our reporter strain maintained characteristics and growth kinetics that were similar to those of the wild-type *E. coli* strain. We found that the intestinal cecum of ICR (CD-1) mice was colonized by O104:H4, with bacteria persisting for up to 7 days after intragastric inoculation. MALDI-TOF analysis of heat-extracted proteins was performed to identify putative surface-exposed virulence determinants. A protein with a high similarity to the aerobactin iron receptor was identified and further demonstrated to be up-regulated in *E. coli* O104:H4 when grown on MacConkey agar or during iron-depleted conditions. Because the aerobactin iron acquisition system is a key virulence factor in *Enterobacteriaceae*, an isogenic aerobactin receptor (*iutA*) mutant was created and its intestinal fitness assessed in the murine model. We demonstrated that the aerobactin mutant was out-competed by the wild-type *E. coli* O104:H4 during *in vivo* competition experiments, and the mutant was unable to persist in the cecum.

**Conclusion:**

Our findings demonstrate that bioluminescent imaging is a useful tool to monitor *E. coli* O104:H4 colonization properties, and the murine model can become a rapid way to evaluate bacterial factors associated with fitness and/or colonization during *E. coli* O104:H4 infections.

## Background

Shiga toxin-producing *Escherichia coli* (STEC) are members of a category of pathogenic *E. coli* that can cause illness ranging from mild intestinal diarrheal disease to severe kidney complications, such as hemolytic uremic syndrome (HUS; reviewed in [1]). Cases and outbreaks of STEC have been associated with the consumption of contaminated food and water. Although more than 100 serogroups have been implicated, the major outbreaks are linked to a very small number of serotypes (reviewed in [2]). In 2011, an uncommon strain of pathogenic *E. coli* serotype O104:H4 caused an unusual number of gastroenteritis and HUS cases, occurring predominantly in adults. The strain originated in northern Germany and disseminated to other European countries [3-5]. The outbreak was originally thought to have been caused by a STEC strain, but was later shown to be produced as a result of an enteroaggregative *E. coli* (EAEC) strain that had acquired the genes for production of Shiga toxins [6-9].

The EAEC category is heterogeneous, and it is associated with cases of acute or persistent diarrhea in children and adults worldwide (reviewed in [10,11]). The virulence of EAEC is known to require a variety of virulence factors. The mechanism by which EAEC exerts pathogenesis; however, is thus far poorly characterized since EAEC strains are recovered from healthy as well as diseased subjects (reviewed in [10,11]). EAEC strains are recognized by their characteristic aggregative or "stacked-brick" adherence pattern and their ability to form biofilms. It has been proposed that host cellular changes during EAEC infection results in digestive-absorptive abnormalities, prolonging the diarrhea [12]. The ability of EAEC to obtain essential nutrients during this process and multiply successfully in this environment is crucial. EAEC, like most bacteria, must acquire iron to survive, since the inability to acquire this metal will disrupt biofilm formation properties and EAEC interaction with human epithelial cells [13]. Therefore, EAEC strains attempting to establish an infection must have the ability to scavenge iron and multiply within the host environment as fundamental requirements for the disease onset.

A wide variety of strategies for acquiring iron have been developed by pathogenic *E. coli*, the most common being the production of siderophores and the utilization of heme [14]. Okeke et al. showed that the most prevalent iron acquisition-encoding genes found in clinical EAEC isolates corresponded to those involved in the synthesis of siderophores, such as in the case of aerobactin [15]. Furthermore, this study found an association between geographical variation of the EAEC strains and their iron utilization genes with disease onset, indicating that most EAEC strains contain more than one iron transport system [15]. There is an urgent need to characterize additional virulence factors in *E. coli* O104:H4, besides the Shiga toxins, which might be associated with disease in the natural setting and not just *in silico* or *in vitro*. Therefore, we combined a murine model that mimics the enteropathogenicity of *E. coli* strains [16,17] with bioluminescent imaging (BLI) technology, a method recently optimized in our laboratory [18]. We hypothesized that the murine model of experimental infection using *E. coli* O104:H4 bacteria not only is an appropriate way to visualize the site of intestinal colonization, but will also aid in rapid screening of putative virulence factors *in vivo*. This BLI infection method provided us with the advantage of quantitatively assessing the *E. coli* O104:H4 burden and facilitated the development of new insights into tissue tropism during infection. Furthermore, BLI application reduced the number of animals required for competition experiments, aided in the localization of *E. coli* O104:H4 infection sites, and enabled us to quickly screen the role of the aerobactin iron transport system (*iut/iuc* system) as a virulence factor in this pathogen.

## Results

### *In vivo* bioluminescence imaging

The *E. coli* O104:H4 *lux* strain RJC001 was generated as described in Methods. We used the pCM17 plasmid containing the *lux* operon under the OmpC constitutive promoter. This plasmid was used for the following properties: to avoid the exogenous addition of luciferase substrate, it carries both a two-plasmid partitioning system and a post-segregational killing mechanism, and maintenance can be ensured for at least 7 days [19]. *E. coli* O104:H4 transformants were plated on the appropriate media, incubated at 37 °C, and monitored for bioluminescence. Colonies that did not display any apparent difference in the bioluminescent signal after patching on plates containing the appropriate antibiotic were further evaluated for their resistance to multiple antibiotics (*E. coli* O104:H4 displayed an extended-spectrum β-lactamase phenotype [20]), presence of multiple plasmids, and growth phenotype similar to that of the wild-type strain (data not shown). *E. coli* strain RCJ001 was selected because it displayed wild-type characteristics and showed a strong bioluminescence signal.

**Figure 1:**
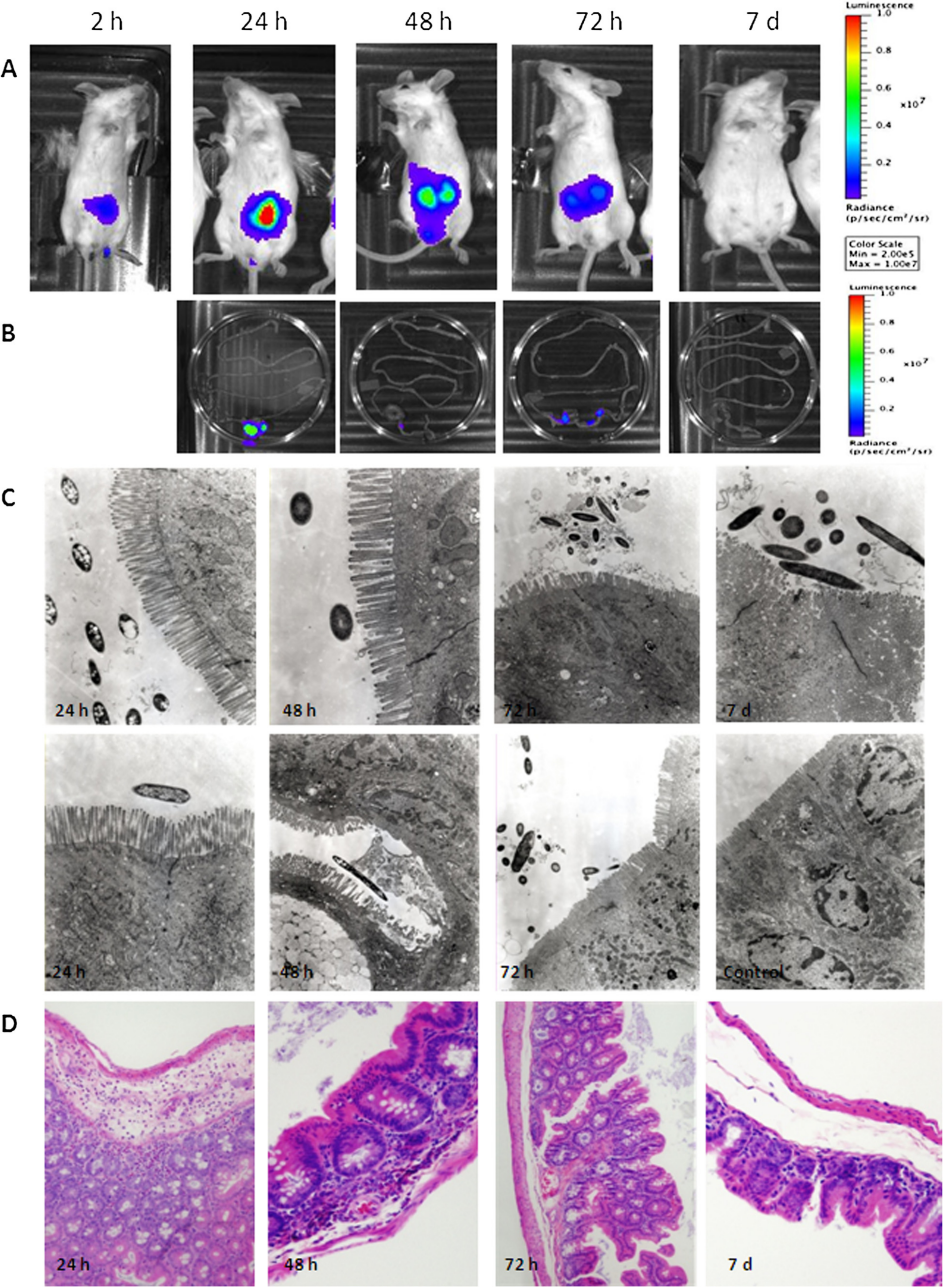
**Bioluminescent imaging characterization and tissue analysis of mice infected with*E. coli*O104:H4*lux*strain RJC001.A**. RJC001 was inoculated via the intragastrical route into ICR (CD-1) mice. The *in vivo* bioluminescence (BLI) imaging was conducted at 2, 24, 48, 72 and 168 h (7 days; 7d) post-infection. The intensity of emission is represented as a pseudocolor image. **B**. At each time point, starting at 24 h, two animals were sacrificed, and intestines were harvested for *ex vivo* imaging and bacterial load determination, and fixed for electron microscopy and histological analysis. Images are representative of 4 replicate experiments. **C**. Ultrastructural studies of the cecum infected with *E. coli* O104:H4 *lux* strain. RJC001-infected cecum demonstrated a slight destruction of the cellular villi and some cell death at 24, 48 and 72 h post infection. Streptomycin-treated, non-infected tissue was used for comparison (control). Magnification corresponds to 31,000-47,000. **D**. Representative images from hematoxylin and eosin-stained mouse cecum at 24 h, 48 h, 72 h and 7 days post infection. Focal inflammatory (PMN) infiltrates in the submucosa were seen at 24 h and 48 h post infection. A couple of sections at 72 h and 7d showed very contained foci of residual necrosis surrounded by normal regenerated tissue, but the remainder of the tissue at the later time points was of normal appearance.

*E. coli* O104:H4 *lux* strain RJC001 was evaluated as a reporter strain in following intestinal infection of the ICR (CD-1) mouse model. A group of 10 ICR mice were infected intragastrically with 1 x 10^8^ CFUs of *E. coli* strain RJC001 (Figure 1A). Every 24 hours, the animals were anesthetized and monitored for bioluminescent signal by using the IVIS spectrum, which collected and quantified the photons emitted by the *E. coli* O104:H4 *lux* infecting the animals. Three animals were sacrificed every 24 hours (except for 72 h and 7 d on which 2 animals were sacrificed), and intestines were harvested for *ex vivo* imaging. Over the course of the study, the bioluminescence signal increased in whole animals, peaking at 24 h and eventually decreasing with time (Figure 1A). The bioluminescent signal was significantly reduced when the intestines were imaged *ex vivo*; however, it was evident that bacteria colonize the murine cecum and persist there throughout the various time points (Figure 1B). A bioluminescent signal was undetectable at 168 h (7 days) post infection. Intestinal cecum sections from different time points were homogenized and plated on LB agar containing kanamycin to determine whether the reporter strain remained in the intestine or was eliminated with time. We recovered 4.8 x 10^6^ ± 1.3 x 10^6^ (at 24 h), 1.6 x 10^7^ ± 4.7 x 10^6^ (at 48 h), 3.2 x 10^7^ ± 9.5 x 10^6^ (at 72 h), and 2.3 x 10^3^ ± 9.7 x 10^2^ (at 168 h) CFUs of strain RJC001, confirming that colonization of the intestinal cecum occurred within 3 days of infection, and lower numbers of bacteria were recovered after 7 days. In our previous work, we reported that the threshold of bioluminescent detection is likely in the range of 1 x 10^3^ - 1 x 10^4^ bacteria [18]; therefore, the low numbers of the reporter strain recovered at 7 days explained the absence of the signal.

### Histological characterization and electron microscopical analysis

We used the remainder of the cecum sections for histological and ultrastructural evaluation. Tissues were processed and examined by electron microscopy to determine whether infection with *E. coli* O104:H4 damaged intestinal epithelial cells. As shown in Figure 1C, bacteria were present in *E. coli* O104:H4-only infected tissues at all time points. Although, no close interaction with the epithelia was observed, destruction of the microvilli and cell death were detected in the sections analyzed at 48 h and 72 h post infection. Macroscopically, the pathological damage of the intestinal wall at these time points was depicted as bleeding upon contact. In contrast, no changes to tissue integrity were observed at 24 h post infection. At 7 days, integrity of the intestinal epithelial barrier recovered, despite an increase in the number of luminal bacteria. The bacteria appeared clustered and surrounded by extracellular matrices of unknown composition, an interesting feature observed at 72 h post infection (Figure 1C).

Histological examination of the H&E-stained infected tissues also revealed scattered inflammatory infiltrates in the submucosa at 24 and 48 h. Inflammatory infiltrates rarely extended to the mucosa and the muscularis. With the exception of rare foci showing residual necrosis and inflammation, the sections collected at 72 h and at 7 days appeared mostly unremarkable (Figure 1D).

### Aerobactin receptor expression is induced on MacConkey agar

**Figure 2:**
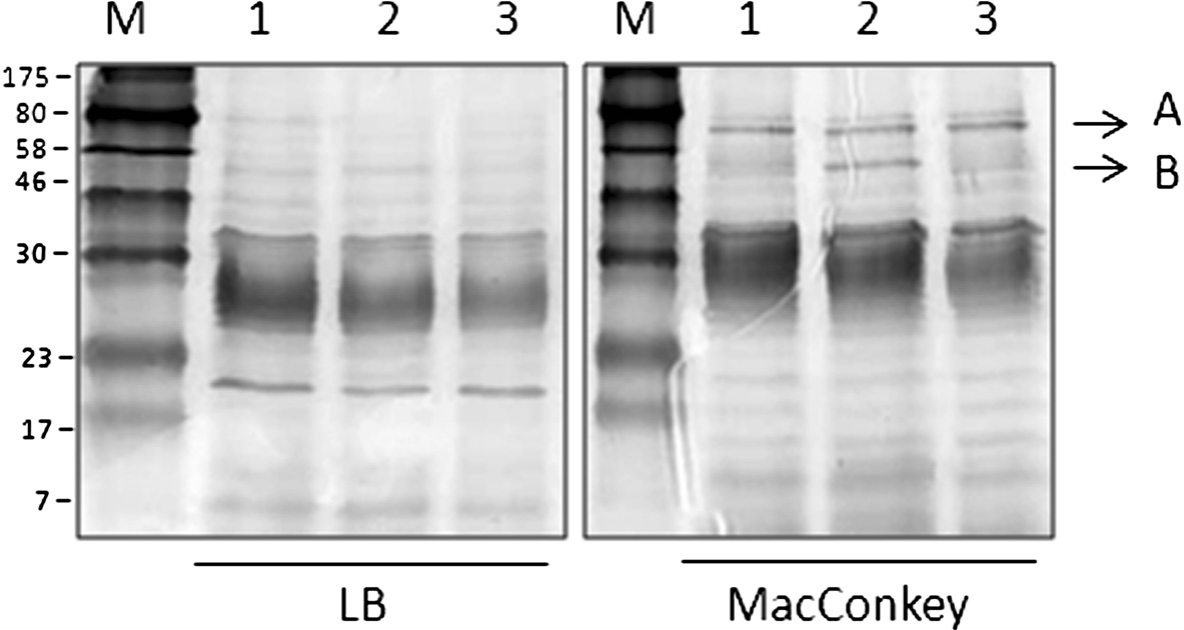
**Detection of differentially expressed surface proteins in*E. coli*O104:H4 strains 15% SDS-PAGE of heat-extracted proteins from*E. coli*O104:H4 strain 2050 (lanes 1), 2071 (lanes 2), and C3493 (lanes 3) grown on LB or MacConkey agar.** The arrows indicate the location of the aerobactin transport receptor (Arrow **A**) and the chain A, dipeptide-binding protein (Arrow **B**).

We have previously demonstrated that expression of novel putative virulence factors, such as the locus for diffuse adherence in atypical enteropathogenic *E. coli*[21] or the enterotoxigenic *E. coli* afimbrial adhesion locus (del Canto et al., manuscript in preparation), are induced when bacteria are grown on MacConkey agar at 37 °C. Furthermore, it is shown that if these factors are expressed on the bacterial surface, a simple extraction method using heat is sufficient in isolating the protein that can then be submitted for sequencing [21]. Therefore, we investigated proteins expressed differentially on MacConkey compared to LB agar in 3 *E. coli* O104:H4 strains: our prototype German *E. coli* O104:H4 isolate C3493 and 2 *E. coli* O104:H4 (strains 2050 and 2071) recovered from an outbreak in the Republic of Georgia. Coomassie-stained SDS-PAGE gel comparison of the heat-extracted protein profiles of the 3 *E. coli* O104:H4 grown in LB and MacConkey agar revealed one protein in all 3 strains with an apparent molecular weight of ~80 kDa when samples were grown on MacConkey agar (Figure 2, protein A). A second protein of ~55 kDa was also expressed in the *E. coli* O104:H4 strain 2071 (Figure 2, protein B). In contrast, these two proteins were absent from the crude heat-extracts of the 3 *E. coli* O104:H4 strains grown in LB agar alone. Both proteins were submitted for MALDI-TOF analysis and identified as the ferric aerobactin receptor (protein A, 731 aa, 80.9 kDa; 18% sequence coverage) and the *E. coli* chain A, dipeptide-binding protein (protein B, 507 aa, 57.4 kDa). The ferric aerobactin transport system is a well-known virulence factor in *E. coli* strains causing extraintestinal infections (reviewed in [22]), such as urinary tract infections [23]. Although its role as a virulence determinant in intestinal *E. coli* is not well understood, it has been proposed that it contributes to the strong colonizing capacity of those strains carrying the aerobactin genes [24]. For this reason, we evaluated the contribution of this iron transport system in the colonization capabilities of *E. coli* O104:H4.

### Low iron concentration in MacConkey induces aerobactin receptor expression

**Figure 3:**
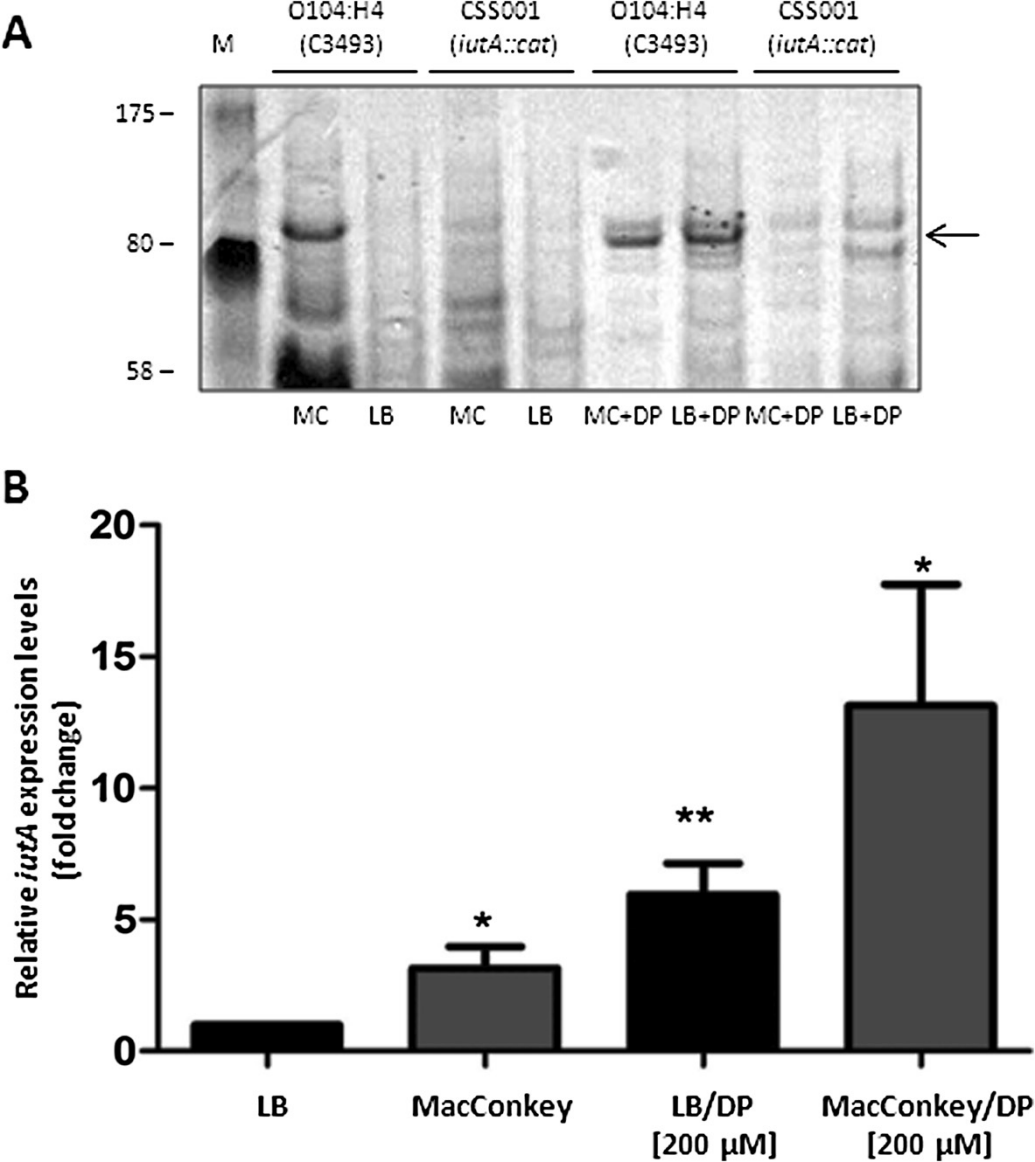
**IutA protein induction and qRT-PCR analysis of*iutA*expression.A**. Heat-extracted proteins of *E. coli* O104:H4 strains C3493 (German isolate) and CCSS001 (*iutA::cat*) grown on MacConkey (MC) or LB agar in the absence (MC or LB) or presence (MC + DP or LB + BS) of 2,2’-dipyridil (DP) were separated in 12.5% SDS-PAGE gels and stained with Coomassie brilliant blue. Molecular mass markers are indicated on the left and the heat-extracted IutA protein is depicted by an arrow on the right. **B**. *E. coli* O104:H4 strain C3493 was grown on LB, MacConkey, LB/DP and MacConkey/DP for approximately 18 h at 37 °C, RNA was extracted and cDNA synthesized. The fold variation of gene expression was obtained by the comparative cycle threshold (∆∆CT) method. The *iutA* expression expressed as a value of 1 represented bacteria grown in LB, and variations in expression in other media conditions are related to this value. The expression of *iutA* resulted in 2.15- (*, P = 0.01), 4.9- (*, P = 0.001) and 12.13-folds (*, P = 0.01), increase in bacteria grown on MacConkey, LB/DIP and MacConkey/DIP respectively. Student’s *T*-test was used for the statistical analysis.

MacConkey agar is considered a low iron-containing medium which has been used to identify high-affinity iron and zinc uptake systems [25]. Therefore, expression of the aerobactin receptor in the *E. coli* O104:H4 wild type and the *iutA* mutant was investigated by using heat-extracted preparations of bacteria grown on agar plates with and without the addition of the iron chelator 2,2’-dipyridyl (DP). Expression was monitored on MacConkey as well as LB agar supplemented with DP, because the addition of the iron chelator is known to induce expression of iron transport systems in *E. coli*[17]. No production of IutA (the 80.9 kDa aerobactin receptor) was observed on Coomassie-stained 12.5% SDS-PAGE gels containing LB agar-recovered bacterial extracts, while abundant IutA was evident in samples from MacConkey plates (Figure 3, panel A). In contrast, the *iutA* mutant lacked detectable expression of IutA on either media tested. To confirm that aerobactin receptor expression responded to iron depletion, the media was supplemented with 200 μM of DP. As shown in Figure 3, panel A, iron chelation resulted in the expression of IutA in bacteria grown on LB + DP as well as MacConkey + DP. As expected, the aerobactin receptor was absent in heat extracts obtained from the CSS001 strain (*iutA::cat*) grown on either of the iron-depleted media. However, for reasons that remain unclear, the expression of the IutA receptor does not appear to be further induced on MacConkey agar supplemented with DP.

Quantitative real-time PCR was performed to support the results obtained with the heat-extracted proteins and to quantify the expression of *iutA* in the *E. coli* O104:H4 wild-type strain, while grown in LB or MacConkey media with and without DP. Basal expression of *iutA* in the wild-type strain was set at a value of 1, and all other values of expression were related to this baseline. The expression of *iutA* was 2.1-fold higher in the wild-type strain grown in MacConkey as compared to LB (Figure 3B, *P* = 0.01). In the presence of DP, the *iutA* expression level in the wild-type strain increased (4.9-fold, *P* = 0.001) when grown in LB + DP and reached 12.1-fold when the wild-type strain was grown on MacConkey agar supplemented with DP (Figure 3B, *P* = 0.01). Overall, data confirmed that the aerobactin receptor is expressed on the surface of *E. coli* O104:H4 wild-type strain, while grown on MacConkey agar, and that expression increased in response to iron depletion.

### Contribution of aerobactin to intestinal colonization

**Figure 4:**
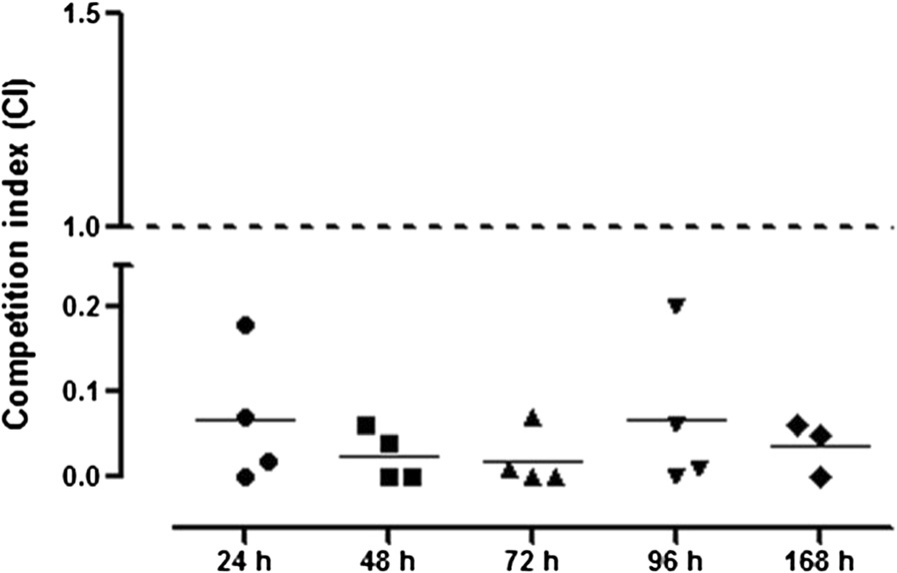
**The*iutA*mutant is outcompeted by*E. coli*O104:H4 strain C3493 in the murine intestine.** Female ICR mice were intragastrically inoculated with 1:1 mixtures of (A) *E. coli* O104:H4 strain C3493 and its isogenic *iutA* mutant CSS001. Animals were euthanized at 24, 48, 72, 96 and 168 h, and the number of colonies recovered from the cecum and counted on antibiotic-containing media was used to calculate the competition index (CI). The CI is the ratio of mutant to wild-type CFU in output samples/mutant to wild type CFU in the inoculum. A CI value of 1 (shown by the black line) indicates that the mutant competes equally with the wild-type strain. Bars represent the geometric mean with the 95% confidence interval. The CIs of samples from the same intestinal site were compared by the Mann Whitney non-parametric test.

Given that the aerobactin transport system has been proposed as a contributor to the strong intestinal colonizing capability of some strains [24], the influence of the mutation of this iron transport system in *E. coli* O104:H4 intestinal colonization in mice was assessed. In a wild-type background, deletion of *iutA* aerobactin receptor gene had a significant effect upon colonization of the cecum (Figure 4). Starting at 24 h post-infection, the wild-type strain outcompeted the *iutA* mutant [geometric mean (95% confidence interval)]; [0.042 (0.01-0.178)]), suggesting that aerobactin production makes a contribution to colonization early during infection. Consistent with the results at 24 h, the CIs of the *iutA* mutant at 48 h [0.047 (0.01-0.183)], 72 h [0.01 (0.01-0.137)], 96 h [0.030 (0.01-0.177)], and 168 h [0.005 (0.01-0.140)]*,* were drastically diminished as compared to the wild-type strain. Data suggested that the *in vivo* intestinal colonization of the *E. coli* O104:H4 strain required the aerobactin transport system, and the defects observed were due to the inability of the strain to acquire iron.

## Discussion

Shiga toxin-producing *E. coli* O104:H4 is a recently identified emerging pathogen that caused an outbreak resulting in a large number of HUS cases and fatalities in adults. Although the serotype O104:H4 was previously isolated in 2001 from a child presenting HUS [9] and in 2006 from a woman who contracted HUS in Korea [26], the unprecedented number of cases, lethality, and complications resulting from the infection identifies this strain as a public threat to human health. The intestinal disease that arises from the *E. coli* O104:H4 causing the outbreak seems to be the result of a hybrid infection that developed from recombination of the Shiga toxin genes from STEC O157:H7 into an EAEC strain, which became evident after sequencing the genome of this isolate [3-5]. Despite the extensive body of literature available regarding STEC and EAEC infections and the study of the pathogenic mechanisms, no data are available on the virulence mechanisms of hybrid strains, as in the case of *E. coli* O104:H4. Data collected by our group and others demonstrated that *in vivo* bioluminescence imaging is a valuable tool for providing insights into mechanisms of pathogenesis, with the goal of identifying new virulence or colonization properties [18,19]. In the current study, it was demonstrated that *E. coli* O104:H4 infection in the streptomycin-treated mouse colonization model can be monitored by using RJC001, a bioluminescent strain of *E. coli* O104:H4.

BLI has been used to study the mechanisms of pathogenesis and treatment efficacies for a number of infectious enteric bacteria. One of the first investigations using BLI was conducted to monitor the virulence differences among strains of *Salmonella enterica* serovar Typhimurium [27]. In that study, the authors showed the utility of the bioluminescence system by visualizing the efficacy of antibiotic treatment in infected animals. BLI in *E. coli* has also been used to track EAEC colonization in the streptomycin-treated mouse intestine [28], and the study proposed that the BLI system offers a simple and direct method to study *in vitro* and *in vivo* competition between mutants and parental strain. Furthermore, the streptomycin-treated mouse colonization model was previously used to investigate the role of other iron uptake systems (e.g. ferrous iron uptake [Feo] system) in *E. coli* K12 [29], and it was demonstrated that iron is an essential source for *E. coli* growth in the murine intestine. It is well known that the maintenance of intestinal colonization requires many properties, among which metabolic competence is of the utmost importance. Therefore, when two strains are in competition for a limited nutrient, like iron, the one that is able to use it more efficiently should outcompete the other [30]. For this purpose, we combined the power of BLI with *in vivo* murine competition experiments to demonstrate that the aerobactin transport system is required for colonization of *E. coli* O104:H4.

The aerobactin transport system is a well-established virulence factor in extra-intestinal *E. coli* infections, but the role of this siderophore system during intestinal infection by pathogenic *E. coli* strains has never been fully established. However, several lines of evidence suggest that this iron transport system might be an important virulence factor for some intestinal pathogenic *E. coli*. A previous epidemiological study performed by our group to identify the distribution of iron utilization genes in collections of EAEC strains isolated during case control studies in Nigeria and Brazil, indicated that the aerobactin transport system is present in >75% of the strains analyzed [15]. Interestingly, a significant association was found between the aerobactin transport and the heme transport systems with more strains from cases than from controls in the Nigerian collection [15]. A recent study has also investigated whether virulence determinants, commonly present in extraintestinal pathogenic *E. coli*, are associated with the fitness of *E. coli* strains in the infant bowel microbiota [31]. The authors found that accumulation of specific sets of virulence markers, including aerobactin and fimbrial adhesin genes in each individual strain [24], correlated positively with its time of persistence in the colon of infant patients. Therefore, they proposed that some bacterial traits contributing to extra-intestinal infections have evolved to increase the fitness of *E. coli* in the intestine [31]. Interestingly, *E. coli* strains that persist and are considered members of the commensal flora can become pathogenic under the appropriate inflammatory conditions in the intestine [32]. For example, members of a newly classified group known as adherent and invasive *E. coli* (AIEC) are commonly found in ileal lesions of Crohn's Disease patients, and they represent isolates that do not have the classical virulence factors found in other *E. coli* pathotypes. Recent studies trying to identify those virulence determinants in AIEC that might contribute to the initiation or persistence of CD indicated that the genome of AIEC strains is closely related to those *E. coli* strains causing extraintestinal infections [17]. Interestingly, all of the AIEC genomes sequenced today possess the aerobactin genes, and it was also demonstrated that iron uptake mediated by the aerobactin system is important for AIEC intracellular survival and mouse intestinal colonization [17]. Overall, the data point to the possibility that the aerobactin transport system participates in the maintenance of the bacteria within the anaerobic environment of the gut. Therefore, this iron transport system in *E. coli* O104:H4 becomes an important “fitness” determinant, as in the utilization of ferric iron, it confers a competitive advantage to this and other pathogenic bacteria over those organisms that do not possess this transport system.

Although the mouse model does not accurately reflect the intestinal infection or complications seen in humans infected with EAEC, STEC or *E. coli* O104:H4, it still remains a relatively practical way to investigate the pathogenesis of *E. coli* strains, especially when compared to more resource-consuming animal models of EAEC/STEC infection, such as the gnotobiotic piglet [33,34] and the rabbit [35,36]. Previous studies have shown that an EAEC O104:H4 strain 55989Str can colonize the streptomycin-treated mouse gut extensively for at least 3 weeks [37]. Even though no sign of disease was evident in the infected animals, the same model was recently used to study the replication of three bacteriophages specific for an EAEC O104:H4 strain, and the mouse intestinal samples enabled the investigators to examine the long-term dynamic interactions between bacteriophages and bacteria within a mammalian host [38]. In the case of STEC, the mouse model has been developed and used to monitor STEC disease and pathology, as well as the impact of Stx in the promotion of intestinal colonization [39]. In our case, the incorporation of BLI analysis proved a useful tool in facilitating the development of an *E. coli* O104:H4 pathogenesis model, as it significantly reduced the number of animals required to identify the intestinal site of *E. coli* O104:H4 persistence and colonization. Although the *lux*-encoded plasmid system that we utilized failed to monitor the infection beyond 7 days and the signal decreased significantly with *ex vivo* intestines, as previously reported [19], it proved to be a useful way of quantifying colonization of this strain while lacking experimental information about putative pathogenic genes. Currently, we are improving our reporter *E. coli* O104:H4 strain by mobilizing a constitutively expressed *lux* operon into its chromosome, providing a stable system that can be used to monitor intestinal colonization and persistence properties for an extended period of time.

## Conclusions

Our findings demonstrate that bioluminescent imaging is a useful tool to monitor *E. coli* O104:H4 colonization properties and present the murine model as a rapid means of evaluating the bacterial factors associated with fitness and/or colonization during *E. coli* O104:H4 infections.

## Methods

### Bacterial strains and mutant construction

All strains used in this study are derivatives of the *E. coli* O104:H4 strain C3493, isolated from a stool sample of a patient with HUS during the 2011 *E. coli* O104:H4 outbreak in Europe. The sample was obtained from the Enteric Diseases Laboratory Branch, Center of Disease Control and Prevention (CDC, Atlanta, GA). Furthermore, 2 *E. coli* O104:H4 strains 2050 and 2071, recovered from an outbreak in the Republic of Georgia, were also obtained from the CDC. Unless indicated, strains were grown overnight in Luria-Bertani (LB) medium at 37 °C, shaking at 225 rpm. The aerobactin transport *iutA* mutant CSS001 was constructed by PCR amplification and cloning of a fragment containing the *iutA* gene, disrupted with the *cam* cassette and cloned into the pCVD442 suicide vector. The mutagenesis approach was previously described [23]. The *iutA* mutant was confirmed by PCR by using the oligonucleotides listed in Table 1, under the following conditions: 1 cycle at 94 °C for 3 min, and then 30 cycles at 94 °C for 1 min, 60 °C for 1 min, and 72 °C for 1 min. For the spatial-temporal location of *E. coli* O104:H4 in mice, the transformed RJC001 was constructed by electroporation with 3 μg of pCM17 plasmid, containing the *luxCDABE* operon driven by the OmpC promoter (constitutive expression), which was previously used to visualize pathogenic *E. coli*[19]. The plasmid was generously donated by J.B. Kaper. Transformants were selected on LB agar plates supplemented with kanamycin (50 mg/ml), and BLI was confirmed by using the IVIS Spectrum (Caliper Corp., Alameda, CA).

**Table 1:** qRT-PCR primers used in this study

**Primer name**	**Sequence**	**Characteristics**	**References**
5RTRRSB	5’-TGCAAGTCGAACGGTAACAG-3’	qRT-PCR *rrsb* gene	[40]
3RTRRSB	5’-AGTTATCCCCCTCCATCAGG-3’		
rpoS Fw	5’-AGTCAGAATACGCTGAAAGTTCATG-3’	qRT-PCR *rpoS* gene	[41]
rpoS Rv	5’-AAGGTAAAGCTGAGTCGCGTC-3’		
iutAFw	5’- GATCATAGTGTCTGCCAGCC-3’	qRT-PCR *iutA* gene	This study
iutARv	5’- GCTCTTTACCGCCCTGAATC-3’		
iutAO104_F	5’-ATGGAGTTTGAGGCTGGCAC-3’	*iutA* mutant confirmation	This study
iutAO104_R	5’-GCTTACTGTCGCTGACGTTC-3’		

### Growth curves

Cultures containing no antibiotics were grown overnight at 37 °C, 225 rpm. On the next day, 1:500 dilutions of overnight were inoculated into 30 mL of pre-warmed, sterile LB media. The growth of CSS001 was compared to the growth of wild-type *E. coli* O104:H4 strain C3493. Sampling was performed at approximately 1-h intervals during the first 9 h of the assay, and a final sample was analyzed 24 h from the start of the experiment. The growth of the *E. coli* wild- type and CSS001 strains was monitored by plating serial dilutions (log_10_ CFU/ml) from the time points on LB media with and without 2,2’-dipyridyl as well as by OD_600_ readings (Additional file 1: Figure S1).

### Mice

Female ICR (CD-1) mice of 20 to 25 g were obtained from Charles River Laboratories and housed in the pathogen-free animal facility at UTMB upon arrival for 72 h prior to experiments. Animal studies were performed in accordance with the Animal Care and Use Committee’s guidelines at UTMB as recommended by the National Institute of Health.

### Bacterial bioluminescence and competition experiments

For the BLI experiments, animals were inoculated with a suspension of RJC001 (*E. coli* O104:H4 *lux;* 1 × 10^8^ CFUs) and, for the competition experiments, with a mixture of *E. coli* O104:H4 wild-type strain and CSS001 (*E. coli* O104:H4 *iutA::cat;* 5 × 10^7^ CFUs per strain) in a final volume of 0.4 ml delivered by gavage (20-gauge needle), thereby using the mouse intestinal model to study enteropathogenicity of *E. coli* strains previously described by our group [16,17]. Briefly, animals received streptomycin (5 g/L in drinking water) for 48 h prior to oral inoculation with the *E. coli* strains and were food restricted for 12 h before oral inoculation. The concentration of the initial inoculum was determined by plating on selective antibiotic LB media by using the dot plate method [42]. Groups of mice (n = 10) were maintained for 7 days, and at different time points (24 h, 48 h, 96 h, and 169 h post-inoculation), groups of two or four animals were euthanized, and the cecum of each animal was collected, weighed, and homogenized for bacterial load enumeration. After homogenization, centrifugation at 3,000 xg for 30 seconds was done in order to sediment the cell debris, allowing for collection of accurate volumes needed to make serial dilutions. Samples were plated on LB agar, LB + streptomycin (100 mg/mL), and LB + streptomycin + kanamycin (50 mg/mL) to determine total bacterial cell counts from those of *E. coli* O104:H4 or the *iutA* mutant strain. The vast majority of bacteria recovered from the cecum corresponded to the O104:H4 isogenic strains (data not shown). The replicates plated for each mouse were averaged, and competitive indices were calculated as previously described [43]. Groups were compared by using the Mann Whitney non-parametric test.

### Bioluminescent quantification

For *in-vivo* imaging, mice were anesthetized with 2-3% isofluorane in an oxygen-filled induction chamber and then transferred to an isolation chamber placed inside the imaging chamber. Bioluminescent images were acquired by using an IVIS Spectrum (Caliper Corp., Alameda, CA) as we previously described [18]. The *ex vivo* images of the intestine were acquired at each time point immediately after euthanasia. Bioluminescent signal is represented in the images with a pseudocolor scale ranging from red (most intense) to violet (least intense) indicating the intensity of the signal. Scales were manually set to the same values for every comparable image (*in-vivo* and *ex-vivo)* to facilitate comparison of intensity of the bioluminescence at each time point.

### Electron microscopy analysis and histopathology

Segments of the mouse cecum infected with the wild-type *E. coli* O104:H4 strain were collected, washed gently with PBS, and fixed in a mixture of 2.5% formaldehyde, 0.1% glutaraldehyde, 0.03% trinitrophenol, and 0.03% CaCl_2_ in 0.05 M cacodylate buffer (pH 7.2) as previously described [16]. Samples were processed further by postfixing in 1% OsO_4_, stained *en bloc* in 2% aqueous uranyl acetate (in 0.1 M maleate buffer, pH 5.2), dehydrated in ethanol, and embedded in Poly/Bed 812 (Polysciences, Warrington, PA). Ultrathin sections were examined in a Philips 201 electron microscope. One observer, masked to the origin of the samples, examined the sections and took random photomicrographs of each sample. For histological analysis, segments of intestinal cecum were instilled with formalin, processed, and paraffin-embedded. Hematoxylin and eosin-stained slides, containing 1–3 sections of cecum, were examined by a pathologist without knowledge of the origin of the specimens.

### Heat-extracted proteins

The strains were grown overnight on LB, MacConkey (Oxoid) agar with or without addition of 200 μM of 2,2’-dipyridyl (DP). The bacterial colonies were suspended in 1x PBS (pH 7.4) and concentrations adjusted spectrophotometrically (600 nm) to 4 x 10^9^ CFU. Bacterial suspensions were incubated at 60 °C for 30 minutes, and then samples were pelleted by centrifugation at 3000 xg for 10 minutes. The supernatant was transferred to a new tube, SDS-sample buffer was added, and samples were boiled at 100 °C for 10 minutes [21]. The samples were separated in 12.5% or 15% SDS-PAGE gels [44]. Expression of distinctively different protein bands were excised from the gel and their identity determined by MALDI-TOF analysis (UTMB Protein core facility) The sequence coverage and the location of the matched peptides are displayed in Additional file 2: Figure S2.

### RNA isolation and cDNA synthesis

Total RNA was obtained from bacteria grown on LB and MacConkey agar with or without 200 μM of 2,2’-dipyridyl; after the bacterial colonies were recovered from the plates and suspended in 4 ml of PBS. The samples were stabilized with RNAProtect reagent (QIAGEN, Valencia, CA) and harvested by centrifugation at 3,500 rpm for 20 minutes. The samples were re-suspended in 10 mM Tris–HCl (in 0.1% DEPC-H_2_O). RNA was purified by using the High Pure RNA Isolation Kit treated with DNaseI (Roche, Mannheim, Germany), quantified, and qualitatively analyzed on 2% agarose gels. Five μg of total RNA was used for cDNA synthesis by the SuperScript First-Strand Synthesis System (Invitrogen, Carlsbad, CA), according to the manufacturer’s instructions. A negative control with no reverse transcriptase was also included. The resulting cDNA was utilized for qRT-PCR.

### Quantitative real-time RT-PCR (qRT-PCR)

The qRT-PCR was performed by using the iQ™ SYBR supermix and the CFX96 System Test (Bio-Rad, Hercules, CA). We used *rrsB* and *rpoS* genes to normalize our data and a value of 1 to standardize *iutA* gene expression in the wild-type strain grown in LB (primers used are listed in Table 1). For each reaction, 1 μl of reverse-transcribed cDNA was subjected to PCR amplification in a 12.5-μl final volume, containing 500 nM of each primer, and 6.5 μl of 2x SYBR supermix. The following conditions were used for amplification: 1 cycle at 95 °C for 30 s, then 40 cycles at 95 °C for 5 sec, and 60 °C for 30 sec. To ensure the specificity of the PCR products, we performed melting curve analysis by heating products from 65 °C to 95 °C in increments of 0.5 °C every 5 s while monitoring the fluorescence. These assays were performed in triplicate for each strain. Student’s *t* test was used for statistical analysis.

## Authors’ contributions

AGT designed experiments and drafted the manuscript. RJC, MRL, CAB, CSS, and RKJ contributed to the conduct of experiments and reviewing the manuscript. ES conducted and provided histological analysis. VLP conducted and provided electron microscopy analysis. NS and JBK contributed with strains and reagents. All authors read and approved the final manuscript.

## Supplementary Material

Additional file 1 **Figure S1. Growth curves of*E. coli*O104:H4 isogenic strains.** Growth curve of wild-type *E. coli* O104:H4 strain C3493 and its isogenic mutant CSS001 (Δ*iutA*) in LB or LB supplemented with 2,2’-dipyridyl (LB + DP) at 37 °C and represented as **A.** CFU/mL and **B.** OD_600_. Click here for file

Additional file 2 ** Figure S2. MALDI-TOF identified peptides matching the aerobactin receptor.** Peptides were identified by MALDI-TOF and subjected to BLAST search analysis which resulted in identification of the Ferric aerobactin receptor precursor from *Escherichia coli* (gi|218692454) with a score 0f 158 and an expected value of 1.5e^-11^. The sequence coverage was 18% and the matched peptides are depicted as bold letters. Click here for file
