# Supplementary figures and images for: In vivo bioluminescence imaging of Escherichia coli O104:H4 and role of aerobactin during colonization of a mouse model of infection
Source: BMC Microbiol. 2012 Jun 20;12:112. doi: 10.1186/1471-2180-12-112 (PMC3438087; doi:10.1186/1471-2180-12-112)

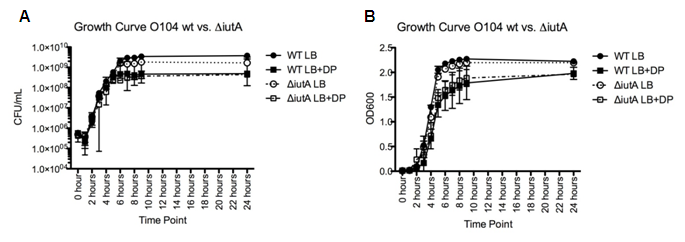

Supplement: Additional file 1 — Figure S1. Growth curves ofE. coliO104:H4 isogenic strains. Growth curve of wild-type E. coli O104:H4 strain C3493 and its isogenic mutant CSS001 (ΔiutA) in LB or LB supplemented with 2,2’-dipyridyl (LB + DP) at 37 °C and represented as A. CFU/mL and B. OD600. [file 1471-2180-12-112-S1.tiff]

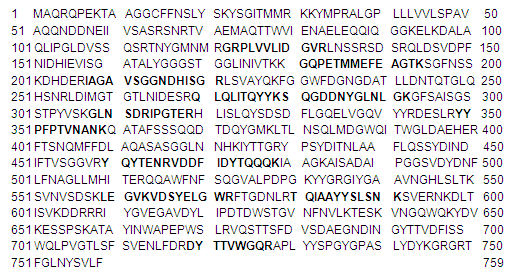

Supplement: Additional file 2 — Figure S2. MALDI-TOF identified peptides matching the aerobactin receptor. Peptides were identified by MALDI-TOF and subjected to BLAST search analysis which resulted in identification of the Ferric aerobactin receptor precursor from Escherichia coli (gi|218692454) with a score 0f 158 and an expected value of 1.5e-11. The sequence coverage was 18% and the matched peptides are depicted as bold letters. [file 1471-2180-12-112-S2.tiff]
